# Pharmacokinetics of dexmedetomidine during analgosedation in ICU patients

**DOI:** 10.1007/s10928-017-9564-7

**Published:** 2017-12-30

**Authors:** Piotr Smuszkiewicz, Paweł Wiczling, Justyna Ber, Justyna Warzybok, Tomasz Małkiewicz, Jan Matysiak, Agnieszka Klupczyńska, Iwona Trojanowska, Zenon Kokot, Edmund Grześkowiak, Wojciech Krzyzanski, Agnieszka Bienert

**Affiliations:** 1 Department of Anesthesiology, Intensive Therapy and Pain Treatment, Clinical Hospital, Poznan University of Medical Sciences, Przybyszewskiego 49 Street, 60-780 Poznań, Poland; 2 Department of Biopharmacy and Pharmacodynamics, Medical University of Gdańsk, Hallera 107 Street, 80-416 Gdańsk, Poland; 3 Department of Clinical Pharmacy and Biopharmacy, Poznan University of Medical Sciences, Św. Marii Magdaleny 14 Street, 61-861 Poznań, Poland; 4 Department of Teaching Anesthesiology and Intensive Therapy, Poznan University of Medical Sciences, Św. Marii Magdaleny 14 Street, 61-861 Poznań, Poland; 5 Department of Inorganic and Analytical Chemistry, Poznan University of Medical Sciences, Grunwaldzka 6 Street, 60-780 Poznań, Poland; 6 Department of Pharmaceutical Sciences, University at Buffalo, 370 Kapoor Hall, Buffalo, NY 14214 USA

**Keywords:** Dexmedetomidine, Pharmacokinetic, Sedation, ICU

## Abstract

Dexmedetomidine (DEX) is a fairly new alfa_2_-agonist which has been increasingly used in recent years for analgosedation, mostly because it offers a unique ability of providing both moderate level of sedation and analgesia without respiratory depression. Despite of many papers published, there are still only a few concerning the PK of the drug given as long-term infusion in ICU patients. The aim of this work was to characterize the population pharmacokinetics of dexmedetomidine and to investigate the potential benefits of individualization of drug dosing based on patient characteristics in the heterogeneous group of medical and surgical patients staying in intensive care unit. This study was performed in the group of 17 males and 10 females patients with a median age of 59.5 years and median body weight of 75 kg. Blood samples for dexmedetomidine assay were collected from arterial catheter, during and after discontinuation of a standard infusion, that ranged from 24 to 102 h. The following covariates were examined to influence dexmedetomidine PK: age, sex, body weight, patients’ health status described by Sequential Organ Failure Assessment Score (SOFA), inotropes usage, and infusion duration. The dexmedetomidine PK was best described by a two-compartment model. The typical values of PK parameters were estimated as 27 L for the volume of the central compartment, 87.6 L for the volume of the peripheral compartment, 38.5 L/h (9.2 mL/min/kg for a 70 kg patient) for systemic clearance and 46.4 L/h for the distribution clearance. Those values are consistent with literature findings. We were unable to show any significant relationship between collected covariates and dexmedetomidine PK. This study does not provide sufficient evidence to support the individualization of dexmedetomidine dosing based on age, sex, body weight, SOFA, and infusion duration.

**Electronic supplementary material:**

The online version of this article (10.1007/s10928-017-9564-7) contains supplementary material, which is available to authorized users.

## Introduction

Dexmedetomidine belongs to non-benzodiazepine drugs often used in intensive care units [1]. It has sedative, anxiolytic and analgesic activity. It is a highly selective alpha_2_-agonist, that is reported to be 8–10 times more selective than clonidine [2]. After intravenous administration its distribution half-life is 6 min and terminal half-life is approximately 2 h [3]. Dexmedetomidine undergoes a complex biotransformation with a small fraction excreted unchanged into urine. The major pathway of biotransformation is glucuronidation, however metabolites are also formed by cytochrome P450 enzymes (mainly 2D6 isoform) [4]. All the metabolites are pharmacologically inactive. Dexmedetomidine is a highly lipophilic drug [5] with a volume of distribution at steady-state of about 118 L and the mean total clearance of 39 L/h [6]. It is also highly (94%) protein bound [7].

Nowadays, more and more attention is paid to ensure the adequate balance between sedation and cooperation with the patient using as few drugs as possible [8]. During intraoperative period it is also important to reduce pain in a way that ensures quick recovery from anesthesia [9]. Dexmedetomidine offers such a unique ability of providing both sedation and analgesia without respiratory depression. The 2013 practice guidelines [10] recommend the use of non-benzodiazepines drugs (dexmedetomidine and propofol) for sedation in intensive care units. It has also been shown that even short oversedation is associated with higher rates of delirium and death. Therefore, the detailed understanding of sedative drugs pharmacokinetics (PK) in various clinical conditions is of special concern. Population modeling is a valuable method to assess pharmacokinetics and pharmacodynamics of drugs in clinical settings as it allows to use sparse and unbalanced blood sampling protocols, that are usual in the ICU. Some studies on dexmedetomidine PK were conducted on a group of healthy volunteers which cannot be simply extrapolated to other patients’ populations [11]. Moreover, it has been reported that dexmedetomidine clearance can increase during long-term infusion [12]. According to the Summary of Product Characteristics [13], there is a risk of both enzymes inhibition and induction due to dexmedetomidine itself, with the clinical significance of this phenomenon being unknown. Both clinical and laboratory data examining the pharmacological properties of DEX are needed to ensure adequate and safe sedation.

The aim of our study was to characterize the population pharmacokinetics of dexmedetomidine in ICU patients during infusion. We also took into account the influence of different covariates on the PK parameters, such as age, sex, body weight, patients’ health status described by Sequential Organ Failure Assessment Score (SOFA), inotropes usage, and infusion duration. Sequential organ failure assessment score (SOFA score) determines the patients organ dysfunction at the admission and/or during ICU stay. SOFA outcome is based on lab results and clinical data related with function of six crucial organs: central nervous, cardiovascular, respiratory, hepatic, coagulation and renal system. Every parameter is estimated in the range 0-4 points. The patient can receive from 0 to 24 points. The SOFA score was found to be a good indicator of prognosis and predicts ICU mortality; score above 11 points indicates probability of death more than 95% [14].

## Methods

### Patients

After approval of protocol by institutional Bioethics Committee, observational study was performed in medical and surgical patients in mixed ICU. The inclusion criteria were age 18 years or older, respiratory insufficiency requiring analgosedation and mechanical ventilation and need for the treatment of hyperactive delirium and agitation refractory to haloperidol in intubated and/or extubated ICU patients. Patients were excluded if they were < 18 years old, heart rate was less than 50 beats per minute and they had significant hemodynamic instability. Patients were evaluated according to the APACHE II score and SOFA score and have measured vital parameters: body temperature, heart rate, systolic, diastolic and mean arterial pressure, central venous pressure and urine output. Dexmedetomidine (Dexdor, Orion Pharma Poland Sp. z.o.o.) was infused continuously without a loading dose. The depth of sedation was determined using the modified Ramsay sedation score to maintain the sedation score of 2–3. The infusion of dexmedetomidine was combined with opioids as needed for analgesia. We started the infusion of dexmedetomidine at the rate of 0.8–1 μg/kg/h that was followed by continuous infusion that ranged from 0.4 to 1.5 μg/kg/h. The infusion of sedative was stopped when there was a significant hemodynamic instability, after the patient extubation or at the discretion of the physician. The supply of the drug was modified according to Ramsay sedation score, and each dose adjustment of dexmedetomidine was recorded. Clinical adverse hemodynamic instability was defined as hypotension (a systolic blood pressure less than 90 mmHg and/or mean arterial pressure less than 65 mmHg) and/or bradycardia (heart rate less than 50 beats/min).

Arterial blood samples (2 mL) for dexmedetomidine assay were transferred into heparinized tubes followed by immediate centrifugation. Plasma was placed in a freezer (− 80 °C). For 22 patients, blood samples were collected just before and at 1, 4, 8, 12, 16, 20 h after initiation of infusion, and then just before and at 5, 10, 20, 60 min and 2, 4 and 6 h after infusion cessation. For 5 patients a slightly modified protocol was used with samples obtained just before and at 2, 8, 24, 32, 48, 56, 72 and 80 h after initiation of infusion, and then just before and at 5, 10, 15, 30, 60 min and 2, 4, 6 and 12 h after infusion cessation.

### Analytical methods

Plasma dexmedetomidine concentrations were determined by LC–MS/MS (liquid chromatography-tandem mass spectrometry) method developed by Szerkus el at. [15] with slight modifications. Briefly analyses were conducted in positive ionization mode. Multiple reaction monitoring (MRM) mode with two transitions for dexmedetomidine and internal standard (detomidine) was used. Dexmedetomidine was monitored at m/z 201.2 → 95.1 and 201.2 → 68.1, whereas detomidine at m/z 187.2 → 81.1 and 187.2 → 54.1. Data acquisition and processing were controlled using Analyst 1.5.2 software (Sciex). The calibration curves were obtained in the range of 0.05–20 ng/mL with correlation coefficient r > 0.995. The analytical procedure was validated, and all steps of the validation confirmed that the applied analytical procedure was suitable for the intended purpose. The within-day coefficients of variation were less than 10%. There were no measurements below the lower limit of quantification.

### PK model

Plasma dexmedetomidine concentrations were described by means of a two-compartment model:

$$V_{C} \frac{{dC_{P} }}{dt} = Infusion(t) - CLC_{P} - QC_{P} + QC_{T} \;\;\;\;\;C_{P} (0) = 0$$

$$V_{T} \frac{{dC_{T} }}{dt} = QC_{P} - QC_{T} \;\;\;\;\;\;\;\;\;\;\;\;\;\;\;\;\;\;\;\;\;\;C_{T} (0) = 0$$

where *Infusion* (*t*) denotes infusion rate of DEX that varies with time, *C*_*P*,_ *C*_*T*_ denote concentrations of dexmedetomidine in central and peripheral compartment, *V*_*C*_ and *V*_*T*_ denote volumes of distribution of the respective compartments, and *CL* and *Q* denote the systemic and the inter-compartmental clearance of dexmedetomidine. Inter-individual variability (IIV) for all PK parameters was modeled assuming a lognormal distribution:

$$P_{i} = \theta_{P} \exp (\eta_{P,i} )$$

where *P*_*i*_ is the individual (for ith subject) parameter, *θ*_*P*_ is the typical value of this parameter in the population, and *η*_*P,i*_ is a random effect for that parameter with the mean 0 and variance *ω*_*P*_^2^. The observed concentration of dexmedetomidine were defined by the following equations:

$${\text{C}}_{\text{P,obs}} = \, C_{\text{P}} (1 + \varepsilon_{prop,C} )$$

where *C*_*P*_ is defined by basic structural model and *ε*_*prop,C,*_ represent the proportional residual random error. It was assumed that *ε* is normally distributed with the mean of 0 and variance denoted by *σ*^2^. The likelihood ratio test for structurally nested models and AIC criterion for non-nested models, the typical goodness-of-fit diagnostic plots, and evaluation of the precision of PK parameter (%RSE < 30 and 50% for fixed and random effects, respectively), variability estimates, and shrinkage were used to discriminate between various models during the model-building process [16, 17].

### Software

Population nonlinear mixed-effect modeling was performed using NONMEM software (Version 7.3.0; ICON Development Solutions, Ellicott City, MD, USA), and the gfortran compiler 9.0. NONMEM runs were executed using Wings for NONMEM (WFN730; http://wfn.sourceforge.net). The NONMEM data processing and plots were done in Matlab Software (Version 9.1; The MathWorks, Natick, MA, USA).

### Covariates search

The main aim of this study was to examine the potential effect of various covariates (weight, age, infusion duration, gender, pretreatment value of SOFA and inotropes usage) on dexmedetomidine PK. The covariate search was performed for parameters with shrinkage lower than 25% [17]. In this analysis the individual estimates of the PK parameters were plotted against covariates to identify their potential effects. If relationship was found, it was described by means of linear regression or power model. The categorical covariates (i.e., gender) were included into the model based on indicator variables.

The significance of potential covariates was systematically evaluated in a stepwise forward selection (ΔOFV < 3.84 points, p < 0.05) followed by backward elimination (ΔOFV < 6.63 points, p < 0.01).

### Bootstrap

Evaluation of model robustness was based on the non-parametric bootstrapping with 1000 replicates. From the bootstrap empirical posterior distribution, 90% confidence intervals (5th–95th percentile) were obtained for the parameters as described by Parke et al. [18].

### Visual predictive check

The model performance was assessed by means of Visual Predictive Check (VPC). The VPC was calculated based on 1000 datasets simulated with the final parameter estimates. The different dosing regimens and variable infusion length required the use of prediction corrected VPC (pcVPC). The pcVPCs were created by correcting the observed and simulated values for the average population prediction in the time-bin divided by population predictions for each observed and simulated value [19–21]. In this study the 10th, 50th and 90th percentile were used to summarize the data and VPC prediction. Since the PK data deviated to some extent from nominal times a binning across time was done.

## Results

**Fig. 1:**
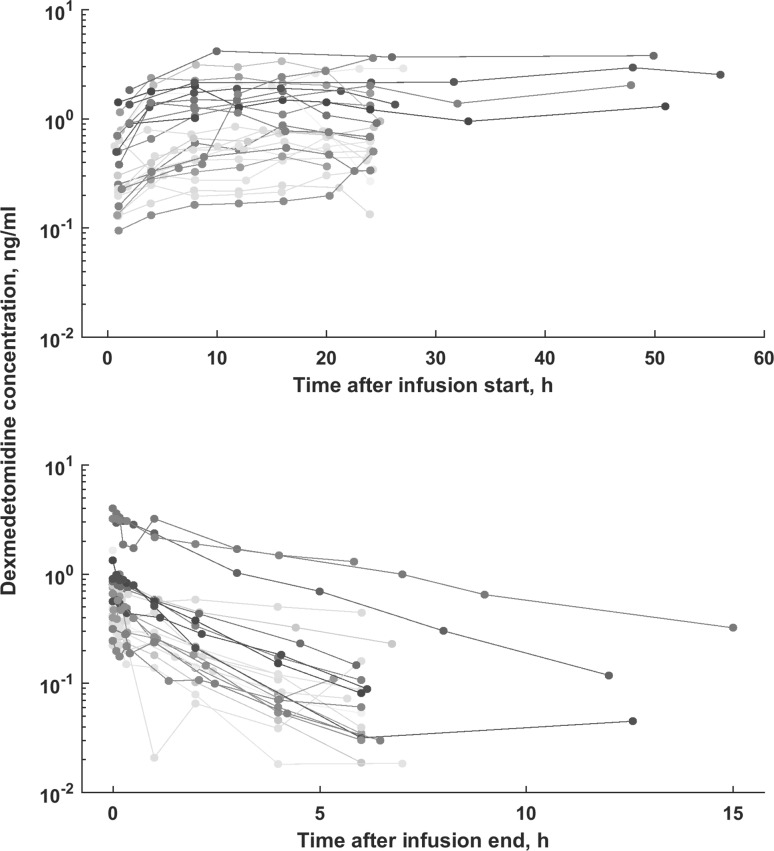
The individual dexmedetomidine concentration–time profiles

This analysis was based on the concentration–time profiles of dexmedetomidine collected from 27 patients (Fig. 1). Table 1 lists the patients’ demographic and the available covariates. The available data consisted of 368 dexmedetomidine concentration measurements (Fig. 1).

**Table 1:** Demographic characterization of patients

Parameter (unit)	Median (range or number)
Age (years)	59.5 (19–84)
Weight (kg)	75 (45–100)
Male/female	17/10
Infusion TIME (h)	42.8 (23.7–102)
Total dose of dex (mg)	1.55 (0.29–6.67)
Infusion rate (μg/kg/h)	0.51 (0.1–1.5)
Use of inotropes (yes/no)	21/6
SYS/DIA (mmHg)	138/65 (60–286/34–155)
MAP (mmHg)	90 (55–152)
HR (bpm)	80 (45–200)
SOFA (scale)	12 (5–16)

Results are expressed as median or range

SOFA (Sequential Organ Failure Assessment) denotes a score to estimate the severity of organ dysfunction and mortality in a potentially septic patient

**Fig. 2:**
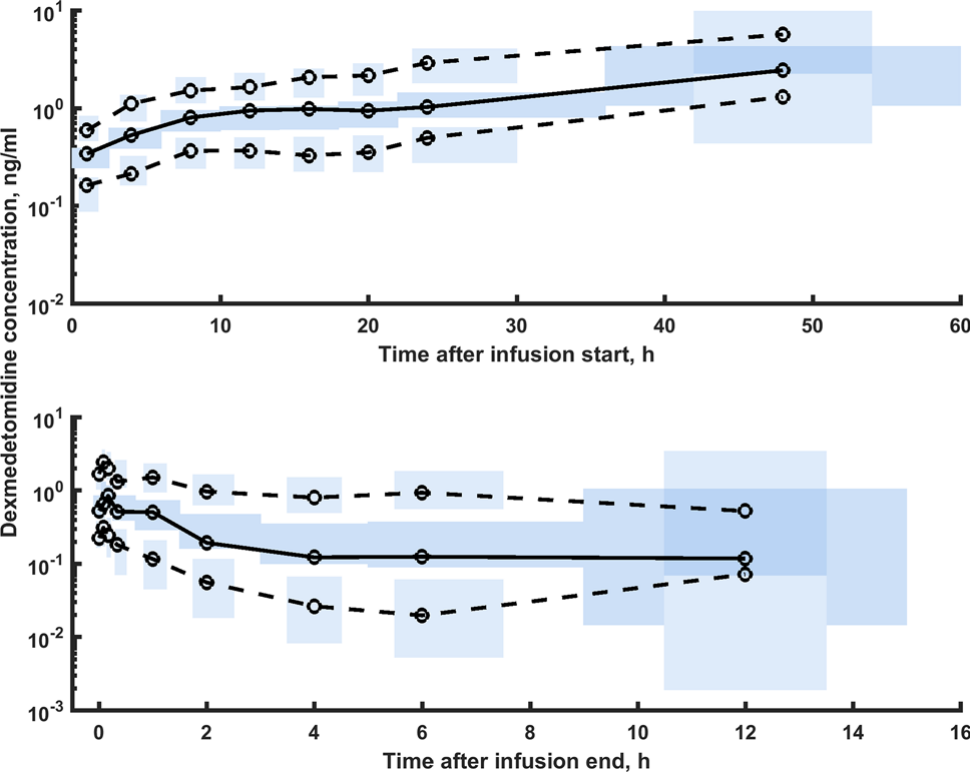
The prediction-corrected VPC plots for dexmedetomidine PK. The VPC plots show the simulation-based 95% confidence intervals around the 10th, 50th, and 90th percentiles of the PK data in the form of blue (50th) and gray (10th and 90th) areas. The corresponding percentiles from the prediction corrected observed data are plotted in black color

The model-building process started with a two-compartment model and proportional residual error model based on literature findings. This model turned out to be the best to describe the data. The simpler one-compartmental model and more complex three-compartment model were not superior based on the applied criteria. Similarly the additive or combined additive and proportional error models did not lead to model improvements based on AIC criterion. Supplemental Fig. 1S shows typical goodness-of-fit plots for the final model. The individual predictions are close to that of the experimental data with no major systematic bias, indicating good performance of the model, which is also confirmed by other goodness-of-fit plots. The VPC for the dexmedetomidine concentration were used to assess the simulation properties of the model. Figure 2 shows the results. VPC plots indicate that both the central tendency of the data and the variability at a particular sampling time were recaptured well. There are no major misspecifications in that graph.

**Fig. 3:**
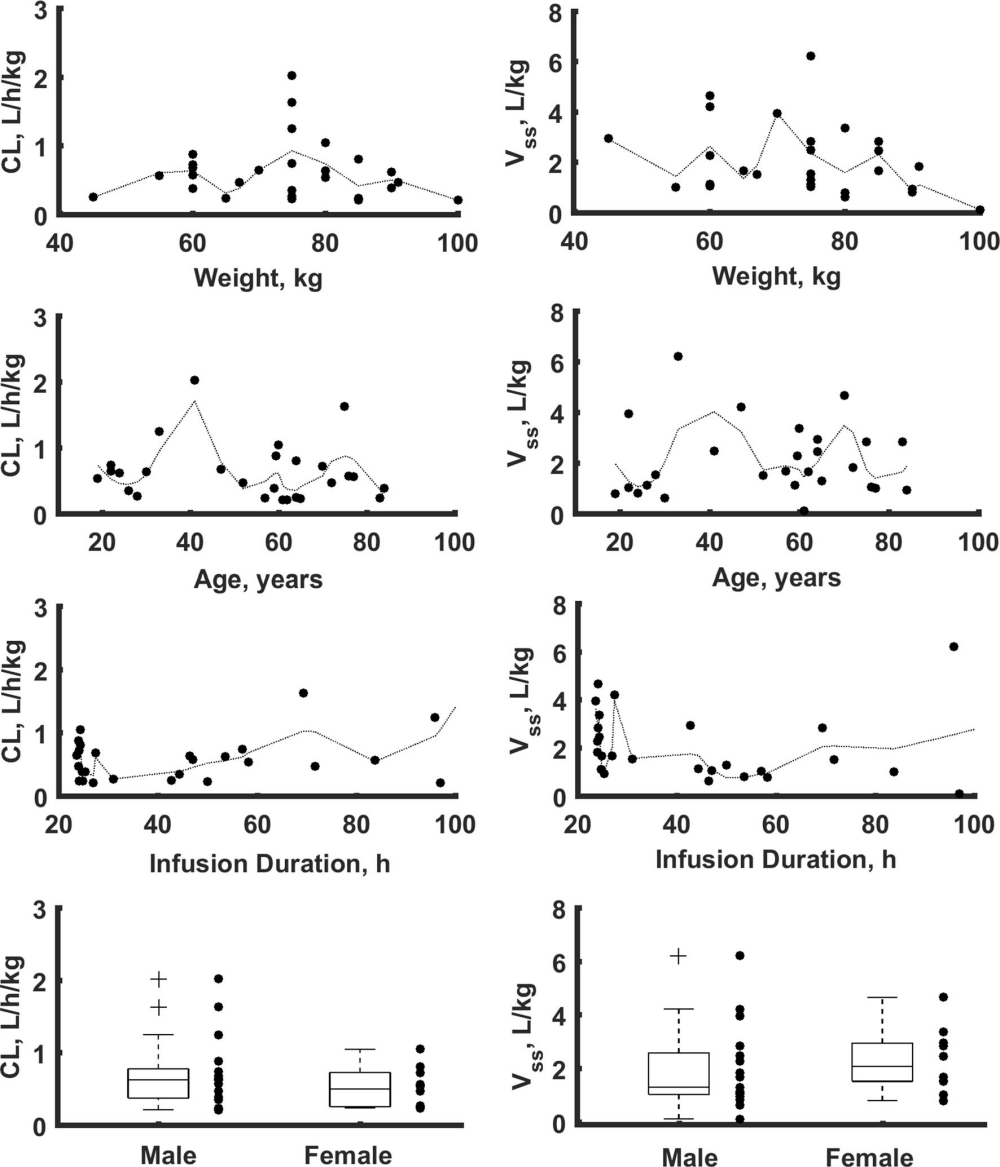
Relationship between body weight normalized clearance and volume of distribution at steady state versus age, body weight, infusion duration and sex for all patients in the study

Table 2 provides the final parameter estimates along with bootstrap results. All parameters, inter-subject and residual error variance were estimated with low (lower than 50%) coefficients of variation (CV). The shrinkage was close to zero for clearance and moderate for other parameters indicating that all the parameters were informative with regard to inter-patient variability and could be used to explore parameter-covariate relationships.

**Table 2:** The parameter estimates of the final PK model of dexmedetomidine

Parameter (unit)	Description	θ, estimate (%RSE) [Shrinkage]	Estimate, bootstrap median (%RSE) [5th–95th CI]
*θ*_*VC*_ (L)	Volume of central compartment	27.0 (31.6)	25.1 (38.6) [12.0–44.8]
*θ*_*CL*_ (L/h)	Systemic clearance	38.5 (12.0)	38.2 (12.1) [32.0–46.8]
θ_*VT*_ (L)	Volume of peripheral compartment	87.6 (17.4)	88.9 (18.8) [65.5–119]
θ_*Q*_ (L/h)	Inter-compartmental clearance	46.4 (25.6)	48.8 (28.8) [28.5–74.2]
Between subject variability			
ω_*VC*_^2^ (%CV)	Inter-individual variability of *V*_*C*_	124 (21.8) [17.6]	117 (29.7) [51.2–162]
ω_*CL*_^2^ (%CV)	Inter-individual variability of *CL*	63.2 (12.0) [0.0]	62.3 (12.2) [49.8–73.8]
ω_*VT*_^2^ (%CV)	Inter-individual variability of *V*_*T*_	89.0 (21.3) [23.6]	85.6 (47.1) [9.0–116]
ω_*Q*_^2^ (%CV)	Inter-individual variability of *Q*	80.9 (25.0) [10.6]	77.7 (29.6) [41.5–115]
Residual error model			
σ^2^ (%CV)	Proportional residual error variability	24 (12.1) [10.2]	23.6 (12.8) [19.3–29.3]

The bootstrap estimates are given for comparison. 25 out of 1000 bootstrap runs terminated early

*RSE* denotes relative standard errors whereas *CV* coefficient of variation

The typical value of the volume of the central compartment (*V*_*C*_) was 27 L (12.0–44.8 L), whereas the volume of the peripheral compartment was higher with *V*_*T*_ equal to 87.6 L (65.5–119 L). The typical systemic clearance (*CL*) and the distribution clearance of dexmedetomidine were 38.5 L/h (9.2 mL/min/kg for a 70 kg patient) and 46.4 L/h. The IIV was estimated for all parameters. It was moderate (63%) for clearance and high (80–120%) for other parameters. There was no clear correlations between the inter-individual random effect estimates.

Possible relationships between patient-specific covariates (age, sex, body weight, infusion duration, sex, SOFA and inotropes usage) and the individual PK parameter estimates were explored graphically (Supplemental Figs. 2S–7S). None of the covariates were found to be statistically significant in this study as there is no clear relationship between them and individual PK parameter estimates. Although the weak correlation coefficient was found for the relationship between infusion length and dexmedetomidine clearance (r = 0.475, with ΔOFV of 6.946, the graphic analysis together with the small number of patients with the infusion duration lasting 4 days and longer did not show any rational reason to include infusion duration as a covariate. The plots showing the relationship between body weight normalized clearance and volume of distribution at steady-state (*V*_*C*_ + *V*_*T*_) versus age, weight, infusion duration and sex are shown in Fig. 3.

## Discussion

We analyzed the pharmacokinetic parameters of dexmedetomidine in adult patients staying 24 h and longer in intensive care unit (the infusion duration ranged from 23.7 to 102 h). We also investigated the patient-specific covariates like age, sex, body weight, SOFA score and infusion duration, concerning their influence on the PK of dexmedetomidine.

Certainly, in the intensive care units the unusual pharmacokinetics might be expected due to the severity of health condition among the patients. To describe the degree of laboratory and physiological abnormalities, different dysfunction scores are used to assess patients’ health status as well as the risk of death. One of these is SOFA which has also been used in previous studies as a potential covariate linked with drugs’ PK and PD [22]. In our study pretreatment values of SOFA score were used as the covariate. The longitudinal changes in SOFA were not considered as they are usually small in this settings for this group of patients.

The obtained PK profiles of dexmedetomidine were best described by a two-compartment model. It is consistent with literature studies, although occasionally (when a drug measurement were available in a late terminal phase of PK profile) a three-compartment model was used [11, 23–29]. The simpler two-compartment model was used to describe dexmedetomidine PK in both healthy volunteers and patients in different health conditions, after single administration or infusions of different durations [11, 25–27].

The typical estimates of volume of distribution at steady state reported in literature are consistent with our findings and range from 79.3 to 161.3 L [11, 27, 28, 30]. Nevertheless, in several studies a very high volume of distribution at steady state was noted (208–389 L). It is likely a consequence of a longer infusion duration (92 h on average) and longer blood sampling after infusion cessation (48 h) than in this study [12, 24]. It suggests that a two-compartmental model likely leads to the biased estimates of volume of distribution at steady state and the observed high variability in volume of distributions across the studies might be contributed to the differences in study designs.

The systemic clearance obtained in this study is in a range of values reported in literature 33.7–53.4 L/h [11, 12, 26–31]. The studies conducted in ICU showed nearly the same typical values of clearance (39 and 39.7 L/h) as estimated in this study [12, 31]. The very interesting phenomenon is the increase of dexmedetomidine clearance with the infusion duration. Iirola et al. presented a case report describing such a relationship. They presented the case of a 42-year-old woman, who received continuous infusion dexmedetomidine for 24 days with maximum dose of 1.9 μg/kg/h. They reported decrease in DEX concentrations by one-third (from 2.9 to 1.7 ng/mL) despite a constant rate of infusion. The calculated clearance increased by 60%. They hypothesized that the cause of increased clearance were changes in hemodynamic variables and reported convergence with the general improvement of the condition of woman [32]. Also in long-term infusion dexmedetomidine in PK study of children population authors described increased clearance during infusion [30]. In our study the infusion durations were quite short (median of less than 2 days), so it was difficult to confirm or reject this hypothesis. We checked infusion duration as a possible covariate and found a week correlation between the length of infusion and dexmedetomidine clearance but due to low number of patients we were unable to confirm or reject the non-stationarity of dexmedetomidine PK during long-term infusions.

The patients included in this study differed according to age (range of 19–84 years), gender (17 males and 10 females), body weight (range of 45–100 kg) and health status. Also a high inter-individual variability in dexmedetomidine pharmacokinetics was observed in this and other studies [33] that warrants the search for covariate relationships explaining this inter-individual differences. In this study several patient-specific covariates including demographic data, patients’ health status and pharmacotherapy (age, body weight, sex, SOFA and the usage of inotropes) were assessed to influence dexmedetomidine PK. None of them was found to be significantly related to individual PK parameter estimates. We are aware that it is rather difficult to establish a covariate relationship when a small number of adults is analyzed or without an a priori assumption of such a relationship. In literature, a clear influence of age and body weight on clearance or body weight alone on clearance was observed in neonates and children [30, 34]. There has been also shown in an animal model that age influences elimination rate constant *k*_*e*_ and PD parametres of dexmedetomidine [35]. In a large group of adult ICU patients undergoing long-term infusion Välitalo et al. found a correlation between body weight and dexmedetomidine clearance that was statistically significant but of no clinical importance [31]. Iirola et al. noted that clearance in ICU patients was not affected by body weight, but it was lower in elderly patients and in patients with low cardiac output [25]. On the other hand, Lin and co-workers claimed an effect of height on dexmedetomidine clearance [24]. It seems that body weight can have an impact on dexmedetomidine dosing, however the true benefits of dosing personalization based on body weight and other covariates are inconclusive and warrant further research.

## Conclusions

The dexmedetomidine PK was best described by a two-compartment model with parameters consistent with literature findings. We were unable to show any significant relationship between collected covariates and PK parameters.

## Electronic supplementary material

Below is the link to the electronic supplementary material.

Supplementary material 1 (DOCX 1294 kb)
