# Supplementary material for: Pharmacokinetics of dexmedetomidine during analgosedation in ICU patients
Source: J Pharmacokinet Pharmacodyn. 2017 Dec 30;45(2):277–84. doi: 10.1007/s10928-017-9564-7 (PMC5845053; doi:10.1007/s10928-017-9564-7)
Supplement: Supplementary file 1 — Supplementary material 1 (DOCX 1294 kb) [file 10928_2017_9564_MOESM1_ESM.docx]

**Supplementary Files:**


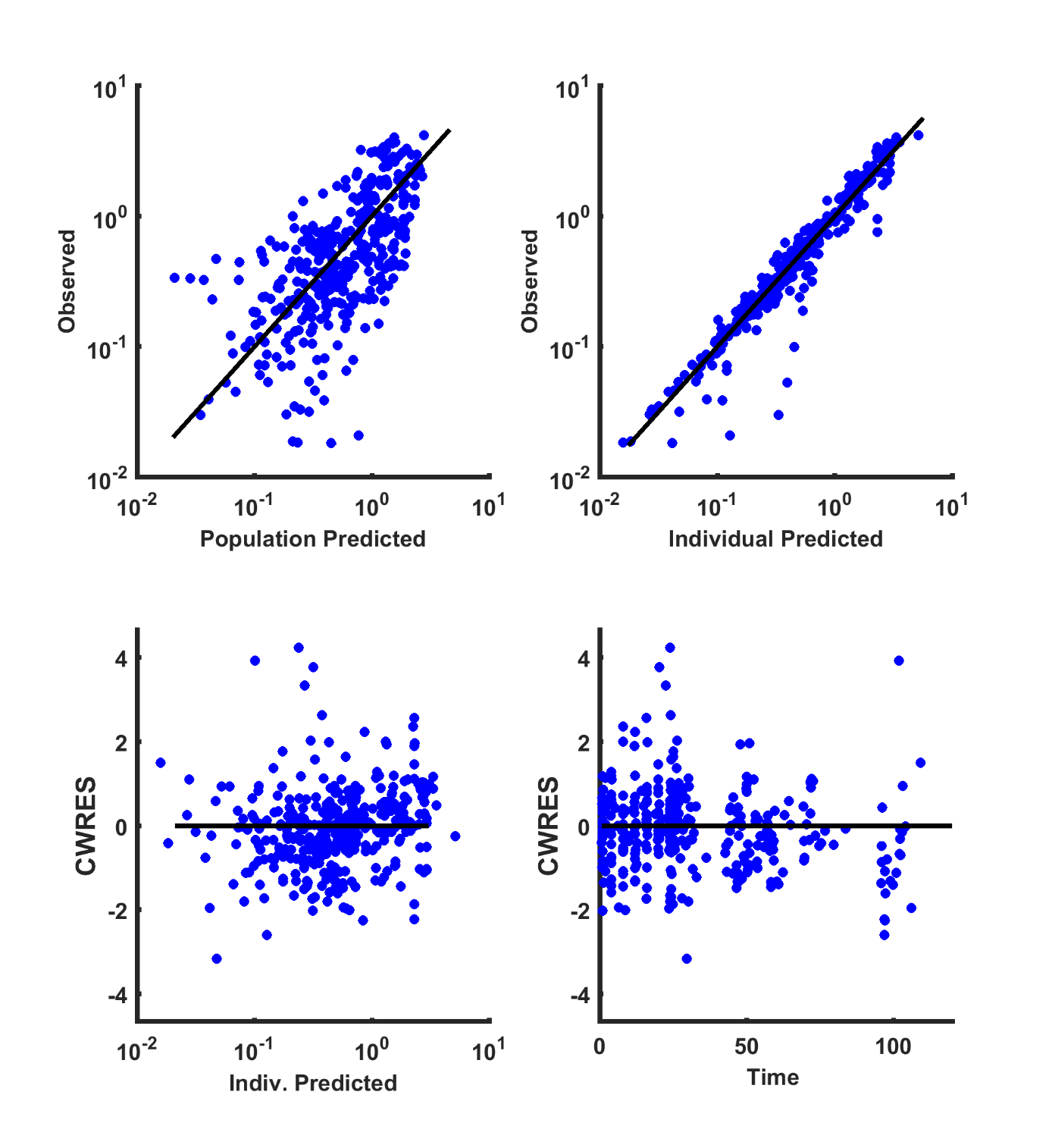


Figure 1S. Goodness-of-fit plots for the final dexmedetomidine PK model: the observed versus the population predicted responses, the observed versus the individual predicted responses, C. the conditional weighted residuals (CWRES) versus the individual predicted responses, and the CWRES versus time.


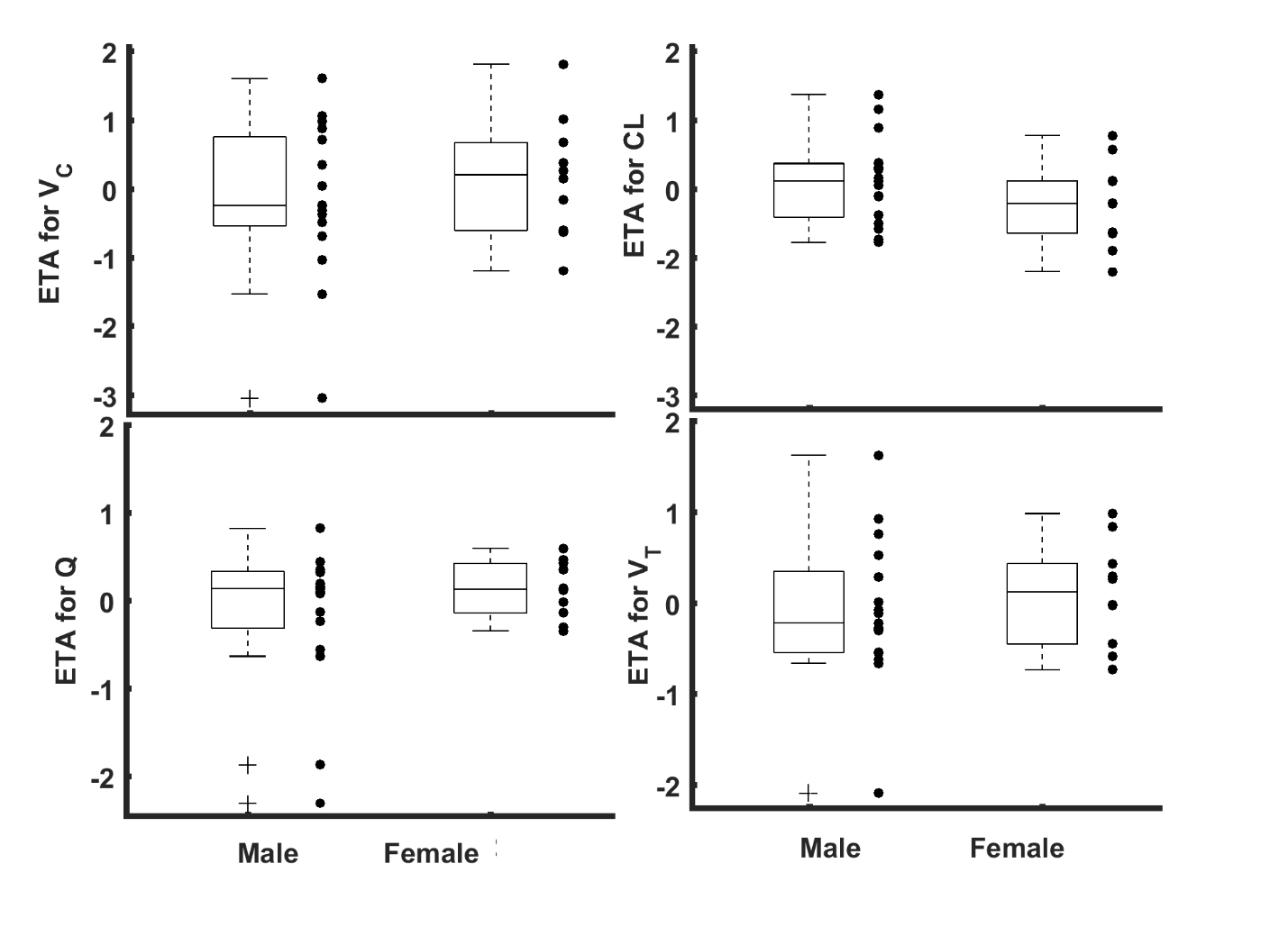


Figure 2S. The individual estimates for ETA (deviation of the individual estimate from the population mean) of dexmedetomidine PK parameters in relation to the sex of the subjects. The box and whiskers plots depict mean, 25th and 75th percentiles, and the most extreme data points excluding outliers (+ symbols).


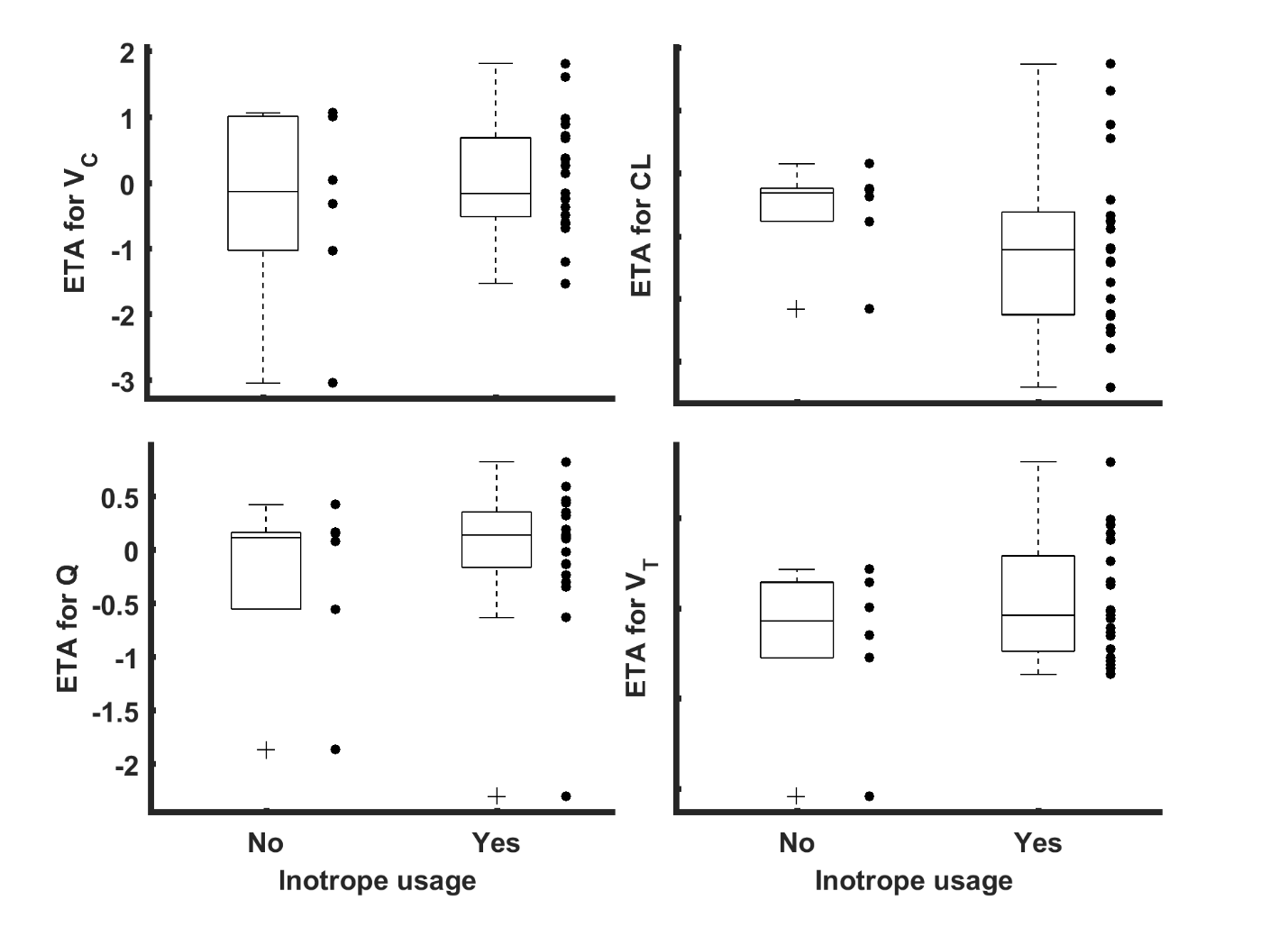


Figure 3S. The individual estimates for ETA (deviation of the individual estimate from the population mean) of dexmedetomidine PK parameters in relation to the subject-specific inotrope usage. The box and whiskers plots depict mean, 25th and 75th percentiles, and the most extreme data points excluding outliers (+ symbols).


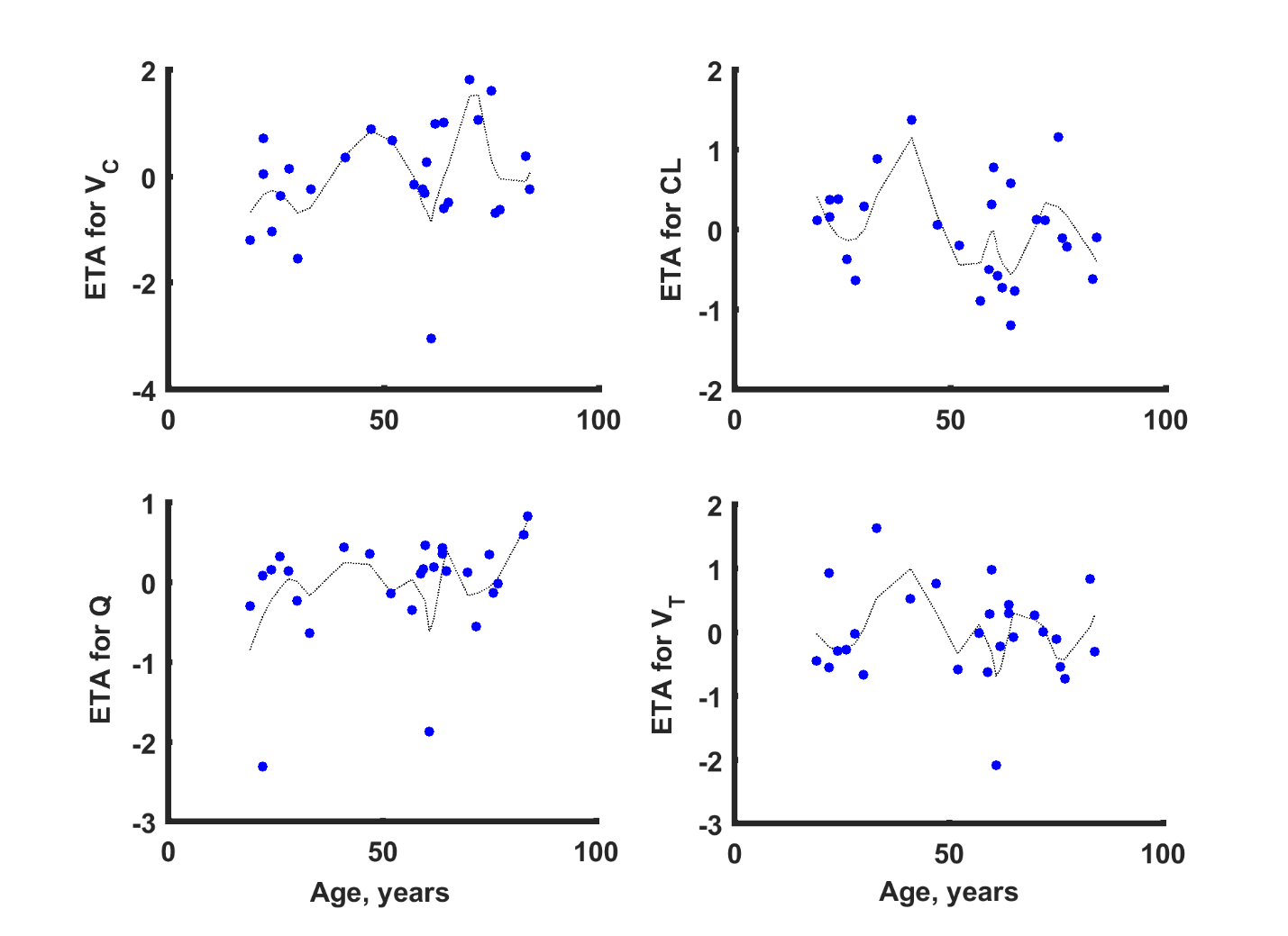


Figure 4S. The individual estimates for ETA (deviation of the individual estimate from the population mean) of dexmdetomidine PK parameters in relation to the ages of the subjects. The lines depict the trend in the data (loess smoothing).


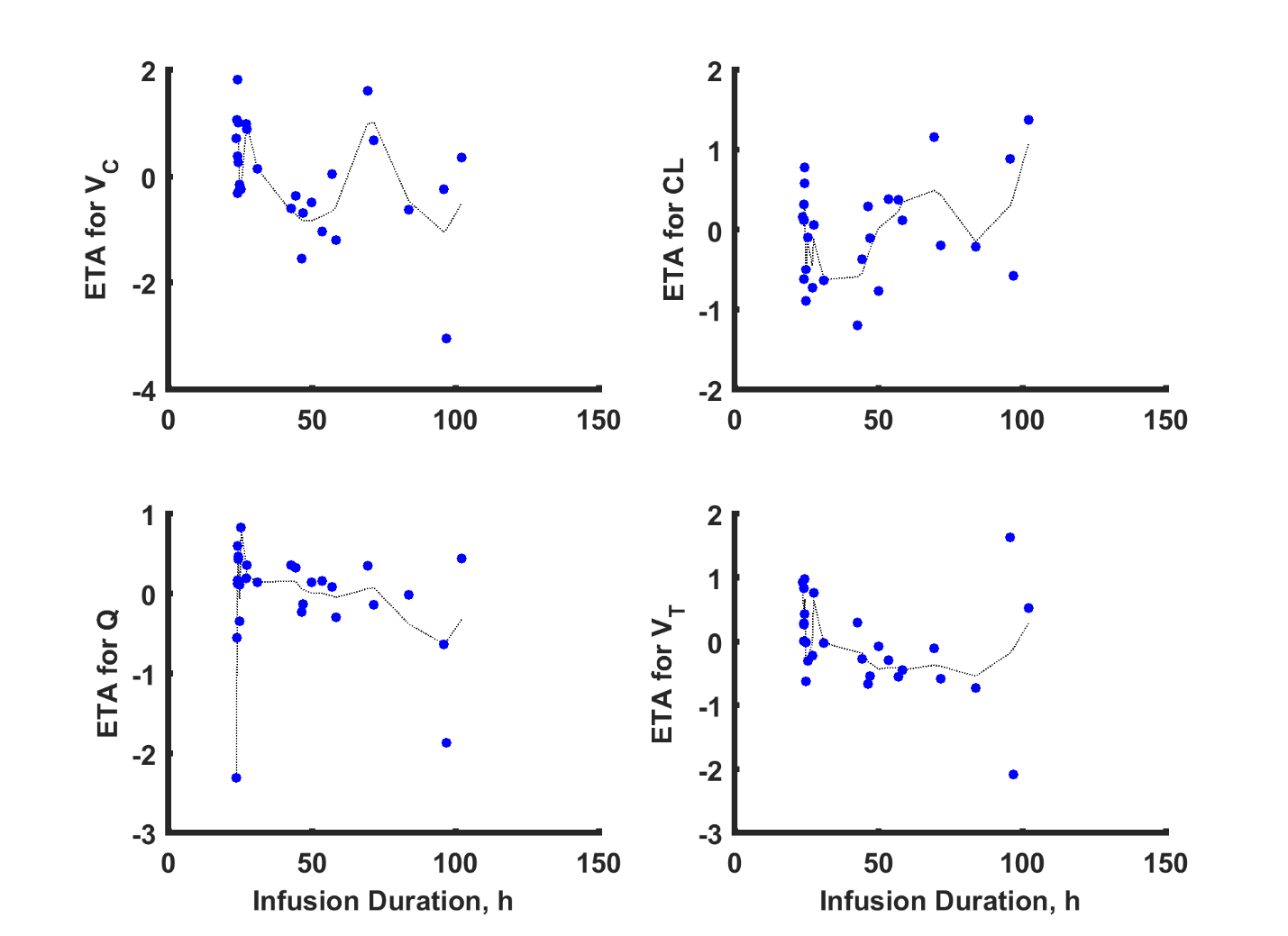


Figure 5S. The individual estimates for ETA (deviation of the individual estimate from the population mean) of dexmedetomidine PK parameters in relation to the subject-specific infusion durations . The lines depict the trend in the data (loess smoothing).


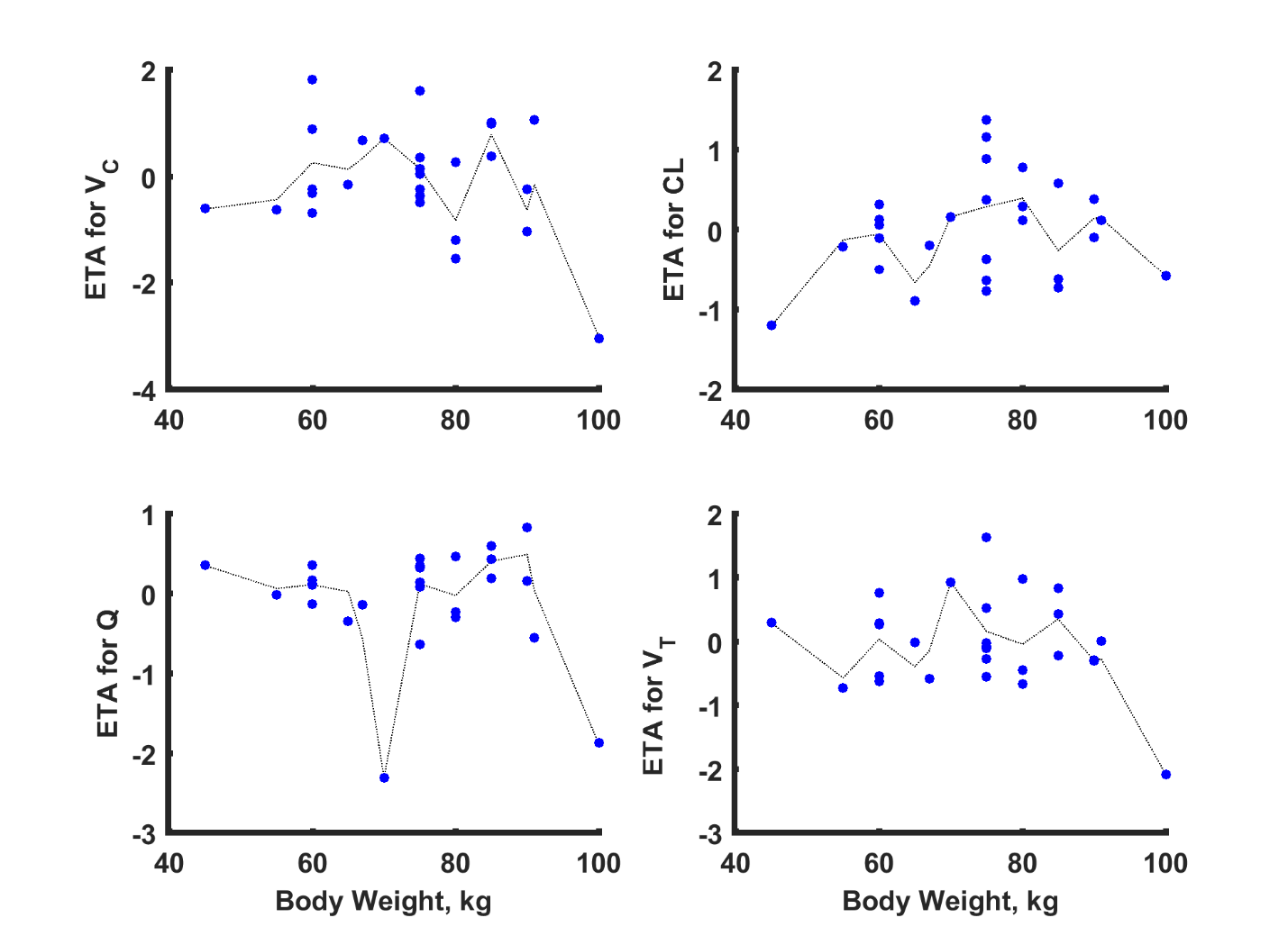


Figure 6S. The individual estimates for ETA (deviation of the individual estimate from the population mean) of dexmedetomidine PK parameters in relation to the body weight of the subjects. The lines depict the trend in the data (loess smoothing).

Figure 7S. The individual estimates for ETA (deviation of the individual estimate from the population mean) of dexmedetomidine PK p
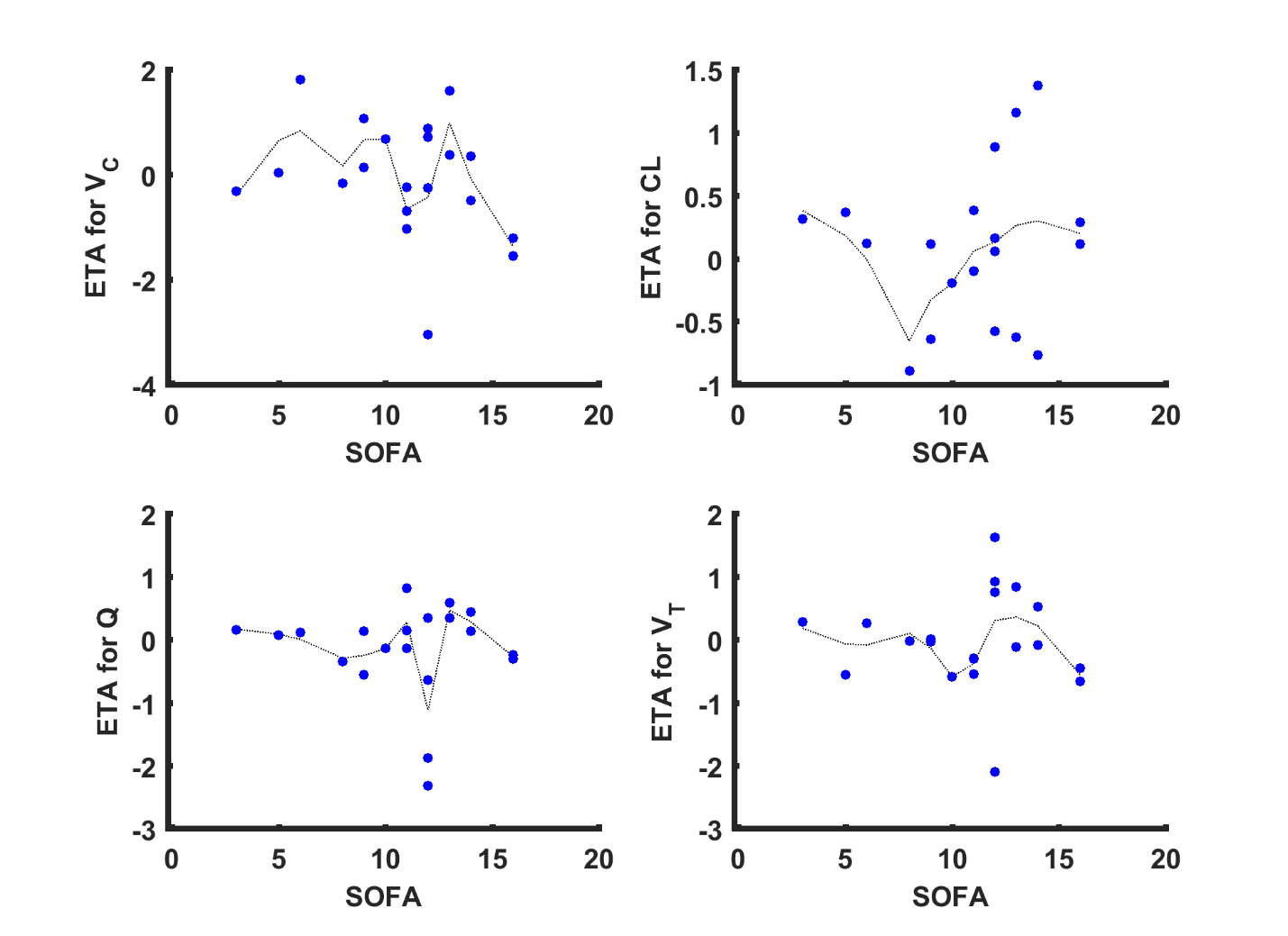
arameters in relation to the pretreatment value of SOFA. The lines depict the trend in the data (loess smoothing).
